# A Gate Hinge Controls the Epithelial Calcium Channel TRPV5

**DOI:** 10.1038/srep45489

**Published:** 2017-04-04

**Authors:** Jenny van der Wijst, Elizabeth H. Leunissen, Maxime G. Blanchard, Hanka Venselaar, Sjoerd Verkaart, Candice E. Paulsen, René J. Bindels, Joost G. Hoenderop

**Affiliations:** 1 Department of Physiology, Radboud Institute for Molecular Life Sciences, Radboud university medical center, Nijmegen, The Netherlands; 2 Centre for Molecular and Biomolecular Informatics, Radboud Institute for Molecular Life Sciences, Radboud university medical center, Nijmegen, The Netherlands; 3 Department of Physiology, University of California, San Francisco, California 94158-2517, USA

## Abstract

TRPV5 is unique within the large TRP channel family for displaying a high Ca^2+^ selectivity together with Ca^2+^-dependent inactivation. Our study aims to uncover novel insights into channel gating through in-depth structure-function analysis. We identify an exceptional tryptophan (W583) at the terminus of the intracellular pore that is unique for TRPV5 (and TRPV6). A combination of site-directed mutagenesis, biochemical and electrophysiological analysis, together with homology modeling, demonstrates that W583 is part of the gate for Ca^2+^ permeation. The W583 mutants show increased cell death due to profoundly enhanced Ca^2+^ influx, resulting from altered channel function. A glycine residue above W583 might act as flexible linker to rearrange the tryptophan gate. Furthermore, we hypothesize functional crosstalk between the pore region and carboxy terminus, involved in Ca^2+^-calmodulin-mediated inactivation. This study proposes a unique channel gating mechanism and delivers detailed molecular insight into the Ca^2+^ permeation pathway that can be extrapolated to other Ca^2+^-selective channels.

The transient receptor potential (TRP) family comprises ion channels with similar structures but with diverse functional properties^1^. TRP channels are composed of four subunits, each containing six transmembrane (TM) segments and intracellular amino (N)- and carboxyl (C)-termini^2^,^3^, thereby resembling voltage-gated potassium (K_v_) and bacterial sodium (Na_v_) channels. Over the last two decades, structural studies on several K_v_ channels have given new insights into channel composition and function^4^. In contrast, research on TRP channel structure-function relation is still in its infancy due to the minimal structural information available.

Within the TRP family, TRPV5 (vanilloid type 5) and its close homologue TRPV6 form a distinct class as the most calcium (Ca^2+^)-selective channel members^5^. TRPV5 constitutes the apical gate for transepithelial Ca^2+^ reabsorption in the kidney and is primarily expressed in the late distal convoluted tubule and connecting tubule of the nephron^6^,^7^. It is characterized as a constitutively active channel, with a substantial Ca^2+^ permeability at physiological membrane potentials^8^,^9^. TRPV5 exhibits a selectivity filter sequence consisting of a ring of four aspartic acid residues (D542) that forms the main extracellular Ca^2+^-binding pocket^10^,^11^. This residue is crucial to many channel characteristics including a high Ca^2+^ permeability, block by magnesium (Mg^2+^), and Ca^2+^-dependent current decay^11^.

The rate of TRPV5 channel inactivation directly correlates with the Ca^2+^ flow through the channel. A high Ca^2+^ level in close vicinity to the intracellular channel mouth functions as a negative feedback mechanism and inhibits TRPV5 channel activity^12^,^13^. Calmodulin (CaM), a ubiquitous Ca^2+^-sensor protein, mediates part of the Ca^2+^-dependent inactivation by binding to the C-terminus of TRPV5^14^,^15^. High intracellular Ca^2+^ levels enhance the CaM binding^15^. Removal of the C-terminal fragment of TRPV5 (S698X) abolishes the sensitivity for CaM, resulting in enhanced Ca^2+^-influx due to decreased Ca^2+^-dependent inactivation^15^,^16^,^17^. Despite these insights, the current knowledge about the gating mechanism of TRPV5 and other Ca^2+^-selective channels at the single molecule level is limited.

The recent elucidation of three TRPV channel structures (TRPV1, TRPV2, and TRPV6) has provided the first insight into channel architecture and possible gating mechanisms^18^,^19^,^20^,^21^. These structures unveil a tetrameric channel topology that has a symmetrical arrangement of four subunits around the central ion conduction pathway. This pathway contains two constrictions (or gates): an upper residue that forms the selectivity filter in the outer pore region and a lower gate at the inner end of TM6^18^,^19^,^20^. These two constrictions were also observed in the recent TRPA1 structure^22^. While the structure of the outer pore domain encompassing the selectivity filter has been well established for these TRPV channels, and likely explain the divergence in TRP channel activation^19^, there is still debate about the lower gate. This is formed by helical bundle crossing of TM6. In TRPV1, this constriction is formed by an isoleucine residue (TRPV1 I679) that is conserved among TRPV members (Fig. 1b)^18^. Mutation of this residue (I679A) resulted in decreased capsaicin currents^23^. A structurally equivalent isoleucine residue also contributes to the lower constriction in TRPA1 suggesting conservation among other TRP channels^22^. A recent study on the TRPV2 structure postulates a methionine as the lower constriction point (TRPV2 M643), which is also shown by a later study demonstrating the TRPV6 crystal structure (TRPV6 M577)^19^,^20^.

In the present study, we aligned this TM6 region in TRPV5 with all TRPV channels and screened for the functional role of the putative restriction points by mutation analysis. In contrast to the postulated lower gate residues, we identify a conserved (in TRPV5/6) tryptophan residue (W583) at the intracellular mouth of the pore that, when mutated, severely affects TRPV5 channel function. In addition, our data demonstrates a conserved glycine hinge in close proximity to W583 that provides flexibility to the lower tryptophan (W583) gate. Furthermore, we reveal crosstalk between the glycine hinge and the C-terminus that is involved in CaM-mediated channel inactivation. The present study provides novel mechanistic understanding of unique structural requirements for TRPV5 channel gating.

## Results

### Comparison of the TRPV channel pores

**Figure 1:**
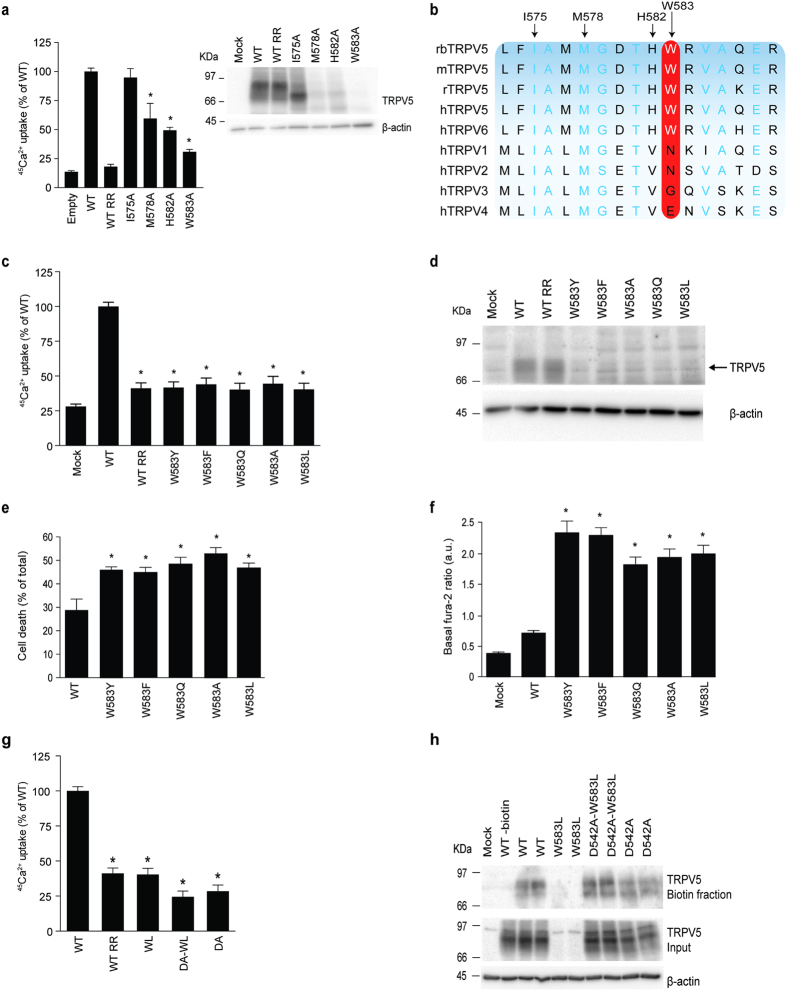
Effect of W583 mutations on TRPV5 function. (**a**) Left panel depicts a ^45^Ca^2+^ uptake assay of HEK293 cells transfected with either wild type (WT) TRPV5, I575A, M578A, H582A, or W583A. Data is shown as percentage of WT (left panel) (N = 9, three independent experiments performed in triplicate). Ruthenium red (RR), 10 μM, is used as control to inhibit the TRPV5-mediated Ca^2+^ uptake. The right panel shows a representative immunoblot using HA antibody for TRPV5 expression, and β-actin as loading control. (**b**) Multiple sequence alignment of the region surrounding W583 in different species of TRPV5 and amongst other TRPV family members. Light blue letters represent conserved amino acids and the red box indicates the conservation of W583. (**c**) ^45^Ca^2+^ uptake assay of HEK293 cells transfected with either wild type (WT) TRPV5 or the indicated mutants, depicted as percentage of WT (upper panel) (N = 9, three independent experiments performed in triplicate). Ruthenium red (RR) is used as negative control. (**d**) Representative immunoblot of cell lysates of the respective Ca^2+^ uptake experiments with HA antibody for TRPV5 and β-actin as loading control. (**e**) Quantification of cell death by counting the number of trypan blue stained (death) HEK293 cells upon transfecting wild type (WT) TRPV5 or the indicated mutants, depicted as percentage of the total cell amount (N = 6, three independent experiments performed in duplicate). (**f**) Basal intracellular Ca^2+^ levels are shown as fura-2 ratio in arbitrary units (a.u.) for HEK293 cells expressing either wild type (WT) TRPV5 or the indicated mutants (N = 30–40, total from two independent experiments). Values are shown as mean ± SEM. Asterisk indicates statistical significance (*p* < 0.05) compared to WT. (**g**) ^45^Ca^2+^ uptake assay of HEK293 cells transfected with wild type (WT) TRPV5 or the indicated mutants, depicted as percentage of WT and corrected for protein expression levels (upper panel) (N = 9, three independent experiments performed in triplicate). Ruthenium red (RR) is used as a negative control. (**h**) Cell surface biotinylation of HEK293 cells transfected with wild type (WT) TRPV5 or indicated mutants. The biotin fraction represents the TRPV5 present at the plasma membrane (top panel) and input demonstrates TRPV5 expression in total cell lysates (bottom panel). Representative immunoblot of three independent experiments is shown.

The flow of Ca^2+^ through TRPV5 is expected to involve two steps. First, Ca^2+^ ions are attracted by the negative charge of D542 at the outer surface of the pore, which serves as the selectivity filter^11^. Second, the lower gate at the intracellular end of the pore region of TRPV5 can determine the influx of Ca^2+^. To determine whether homologous residues of the postulated lower gate (I575 and M578) play a role in TRPV5 Ca^2+^ permeation, we evaluated channel function of I575A and M578A mutants by ^45^Ca^2+^ uptake assay. The I575A mutant did not show altered channel expression or function (Fig. 1a), which was expected if I575 is considered the channel gate. The M578A mutant did not demonstrate complete loss of function, pointing towards involvement of additional residues in the lower gate. As TRPV5 exhibits a unique high Ca^2+^-selectivity, its channel gating might be differently regulated compared to other TRPV channels. Interestingly, TRPV5 contains a histidine residue (H582) and tryptophan residue (W583) that are highly conserved among different species and in TRPV6, but not in other members of the TRPV family (Fig. 1b). The ^45^Ca^2+^ uptake assay demonstrated a significantly reduced uptake of H582 and W583 compared to wildtype TRPV5. Given the hydrophobic nature of the latter amino acid, we hypothesized that the position of this bulky amino acid might be energetically unfavorable for ion permeation depending on side chain conformation.

### Mutation of W583 increases the Ca^2+^ permeability of TRPV5

Site-directed mutagenesis was applied to substitute W583 for amino acids with different characteristics (aromatic: W583Y and W583F; non aromatic: W583Q, W583L and W583A). The activity of the mutant channels was first assessed by a ^45^Ca^2+^ uptake assay. Ruthenium red (RR) was used to measure the TRPV5-mediated component of the ^45^Ca^2+^ influx^24^. A significantly diminished ^45^Ca^2+^ uptake was observed in all mutants compared to wild type TRPV5 (Fig. 1c). However, TRPV5 protein expression was also strongly reduced in cells expressing the different mutants (Fig. 1d). This results from an observed decrease in cell survival, which is confirmed by a trypan blue assay demonstrating significantly increased cell mortality for all mutants compared to wild type TRPV5 (Fig. 1e). To establish a link between enhanced Ca^2+^ uptake and reduced cell survival, the basal intracellular Ca^2+^ levels were measured using fura-2-AM, a cell-permeant ratiometric fluorescent Ca^2+^ indicator. Using this strategy, a significant increase in baseline Ca^2+^ levels was detected in cells expressing mutant TRPV5 (Fig. 1f).

To demonstrate that the observed cell death is a result of increased TRPV5-mediated Ca^2+^ influx, we mutated the selectivity filter (D542A) that is known to ablate Ca^2+^ permeation of the channel^11^. The ^45^Ca^2+^ uptake of D542A/W583L was not different from D542A, confirming the block of Ca^2+^ permeation (Fig. 1g). In contrast to previous experiments, the ^45^Ca^2+^ uptake was corrected for protein expression in order to demonstrate differences in TRPV5 activity since the expression of the single mutant W583L was nearly absent on the immunoblot, while the double mutant D542A/W583L is expressed at similar levels as wild type TRPV5 and D542A (Fig. 1g). To demonstrate channel expression at the plasma cell membrane, a cell surface biotinylation was performed using cells expressing wild type TRPV5, or either single or double mutants. Similar to Fig. 1d, the overall protein expression (in both biotin and input fraction) of W583L was severely diminished. This was rescued by the D542A mutation since the double D542A/W583L mutant showed equal plasma membrane abundance to wild type TRPV5 and D542A (Fig. 1h).

### Mutation of W583 affects the intrinsic channel properties of TRPV5

**Figure 2:**
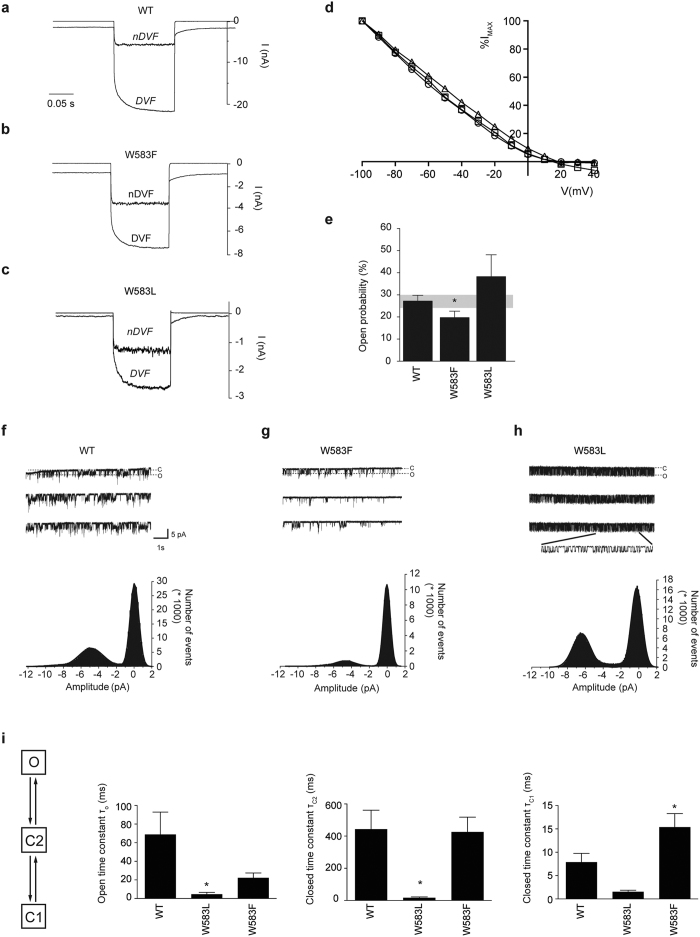
Channel activity is altered in the TRPV5 W583 mutants. (**a**–**c**) Representative traces of the whole-cell Na^+^ currents at the voltage step to −100 mV of HEK293 cells expressing either wild type (WT) TRPV5 or the indicated mutants (W583L and W583F) in nominally DVF (nDVF) and divalent-free (DVF) solutions. (**d**) Representative current-voltage relationship of wild type (WT) TRPV5 (circle), W583F (triangle), and W583L (square) measured from a voltage step protocol of −100 to + 40 mV in the whole cell configuration in DVF solution and current is depicted as percentage of the maximal current at −100 mV (%I_max_). (**e**) Histogram showing the average open probability at −80 mV for wild type (WT) TRPV5, W583F, and W583L. Values are shown as mean ± SEM (N > 7 per condition). Asterisk indicates statistical significance (*p* < 0.05) compared to WT. (**f**–**h**) Cell-attached single channel recordings were measured during a 10 s step to −80 mV (holding potential 0 mV) in wild type (WT) TRPV5 (**f**), W583F (**g**), and W583L (**h**). Bottom panels show the amplitude histograms with a Gaussian fit function corresponding to the closed and open states in the upper panels. (**i**) The dwell time constants (τ) are extracted via a three-state model of one open (**o**) and two closed states (C1 and C2). Average τ values of wild type (WT) TRPV5, W583F, and W583L were derived by two-exponential fit of the model-based distribution of dwell times, obtained from single channel recordings (N > 7). Values are shown as mean ± SEM. Asterisk indicates statistical significance (*p* < 0.05) compared to WT.

Next, a non-aromatic and an aromatic TRPV5 mutant (W583L and W583F) were subjected to whole-cell patch clamp recording to examine their channel characteristics using a voltage step protocol (from −100 to 40 mV). Figure 2a–c show representative traces of wild type TRPV5, W583L and W583F at the voltage step to −100 mV. The current-voltage relationship is established from the voltage step protocol and is similar between mutant and wild type TRPV5 (Fig. 2d). Importantly, the mutant channels exhibit the characteristic Na^+^ inward-rectifying current in nominally divalent-free (nDVF) bath solution, which is enhanced upon addition of EGTA-containing solution (DVF, 100 μM EGTA) (Fig. 2a–c). The overall deleterious effect of mutant TRPV5 expression in HEK293 cells did not allow for accurate quantification and comparison of current densities between mutant and wild type TRPV5. Of note, the absolute currents of both mutants were 3–4 fold smaller than wild type TRPV5.

In order to assess putative changes in the intrinsic properties of the mutant TRPV5 channels, cells expressing wild type or mutant TRPV5 were subjected to cell-attached patch clamp. Briefly, TRPV5 activity was measured by applying a series of 10 s voltage steps to −80 mV from a holding voltage of 0 mV, using Na^+^ as the charge carrier. By applying this protocol, frequent patches containing one to three channels were obtained and analyzed for amplitude of openings and open probability. Representative channel openings and amplitude histograms of wild type, W583L and W583F are depicted in Fig. 2f–h. While the aromatic W583F showed a decreased open probability compared to wild type TRPV5, the non-aromatic W583L mutant presented a tendency of increased single channel activity (Fig. 2e). Analysis of other mutant recordings also revealed a trend towards increased open probability for W583Y, W583A, W583Q (significant) compared to wild type TRPV5 (Fig. S1A,B). The amplitude of opening at −80 mV was not significantly different between wild type TRPV5 and the mutants (Fig. S1C).

In order to provide more insight into the kinetics of the single channel behavior, we compared wild type, W583F and W583L in our model-based fitting of the duration histograms and subsequent dwell-time analysis of the open and closed states. We hypothesized a three-state model (O ↔ C2 ↔ C1): an open state (O), closed state (C1), and pre-open closed state (C2), as detailed analysis demonstrated that one open and two closed exponential components are required to describe the open and closed dwell-time distributions. The average time constants of the open and closed states were significantly reduced in W583L compared to wild type TRPV5, which indicates that the channel switches faster between states (Fig. 2i). This was confirmed by a significantly increased closing rate constant for W583L (Fig. S1D). The W583F mutant was not significantly different from wild type TRPV5 apart from an increased time constant in the closed state (C1) that could explain the decreased open probability (Fig. 2e,i).

### Detailed insight into the TRPV5 pore

**Figure 3:**
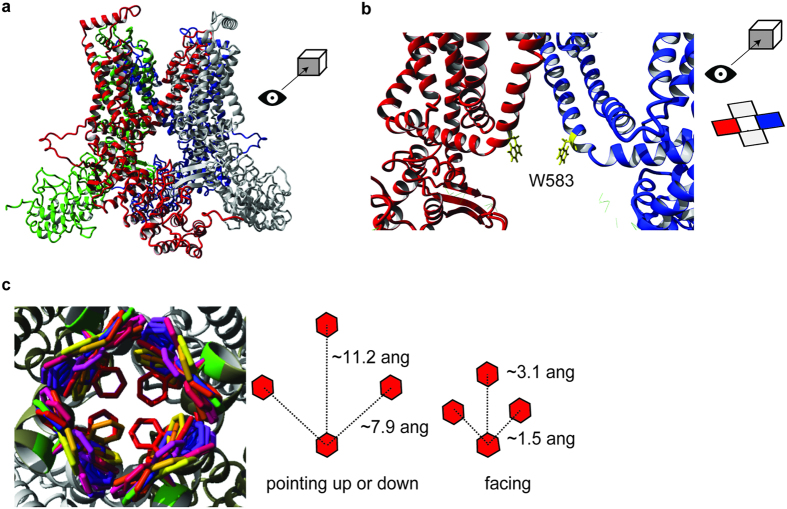
Homology model of TRPV5. (**a**) The tetrameric TRPV5 structure is depicted in front view. Each monomer is color-coded. (**b**) The detailed front view of the pore region highlights the side chains of W583 (yellow) in two monomers. The side chains are sticking towards the permeation pathway. (**c**) The possible rotameric positions for the side chain of W583 are shown in different colors (left panel). Overall, three main rotameric positions are detected with the side chains either pointing upwards, downwards or pointing towards each other. The distance between the W583 side chains is based on the homology model and is depicted for the main rotameric positions (right panel).

To investigate a structural basis for TRPV5 channel gating, we built a homology model of TRPV5 using the recently determined structure of rat TRPV1 (16) (Fig. 3a, Fig. S2A). According to the Sander-Schneider plot, the 29% identity between rabbit TRPV5 and rat TRPV1 is sufficient to perform homology modeling^25^. The model represents the tetrameric structure of TRPV5 with the putative transmembrane core surrounding a central cavity for ion permeation. First, the molecular surface representation shows the localization and orientation of the D542 residue as a negatively charged ring at the entry of the channel (Fig. S2B)^11^,^26^. Interestingly, the inner pore region contains the tryptophan residue (W583) residue at the intracellular end of TM6, with its side chain pointing towards the ion permeation pathway (Fig. 3b and d, Fig. S2B,C). Given the hydrophobic nature of the water facing tryptophan side chain, this conformation appears energetically unfavorable. However, the side chain of a tryptophan can convert to different rotameric positions, which often depends on the backbone conformation^27^. The YASARA & WHAT IF Twinset was used to extract the preferred rotamers and the associated probabilities, depicted as different colors in Fig. 3c (left panel). The three main rotameric positions, with the side chains either facing up, down or towards each other, demonstrate a considerable degree of variation in ion permeation space. When the W583 side chains are facing each other, the estimated distance between them is ~3.1 Å, and therefore, the estimated passage size is around 0.8 Å, while the other two rotamers form a wider pore (Fig. 3c).

### G579 forms an inner pore helix hinge in TRPV5

**Figure 4:**
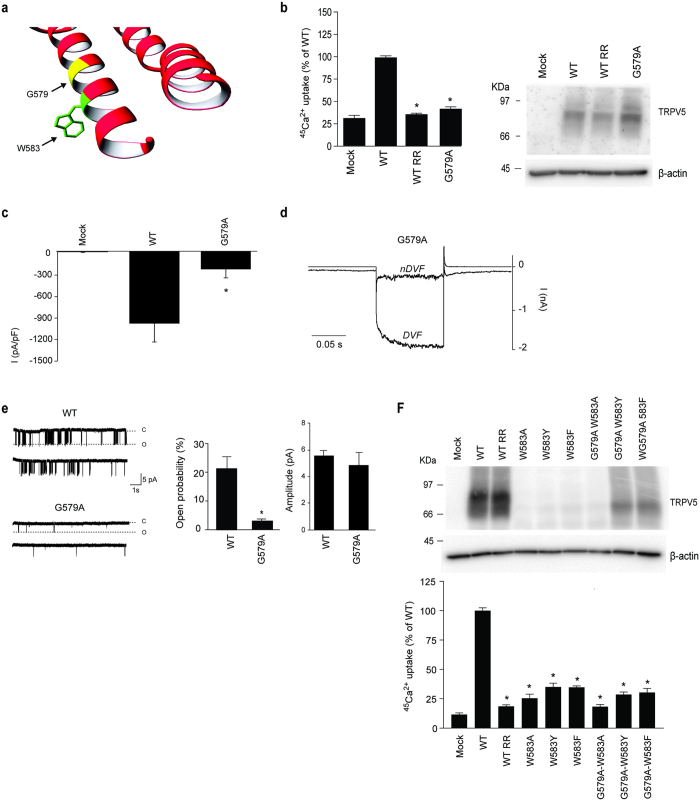
Analysis of the G579A mutation on TRPV5 activity. (**a**) Zoomed image of the α-helix containing W583 and G579. (**b**) ^45^Ca^2+^ uptake assay of HEK293 cells transfected with either wild type (WT) TRPV5 or the G579A mutant. Ruthenium red (RR) is used as control for TRPV5-mediated uptake. The protein expression level is shown in the representative immunoblot (N = 9, three independent experiments performed in triplicate). (**c**) Averaged Na^+^ current densities of mock, wild type (WT) TRPV5 or G579A transfected HEK293 cells are presented at −80 mV in DVF solution (N = 8–10). (**d**) Representative traces of the whole-cell Na^+^ currents of HEK293 cells expressing TRPV5 G579A in nDVF and DVF solutions, in response to a voltage step protocol (−100 to +40 mV). (**e**) The open probability at −80 mV is assessed using cell-attached patch clamp in cells expressing the G579A mutant. Typical single-channel behavior is shown for wild type (WT) TRPV5 and G579A. The average open probability (middle panel) and amplitude (right panel) are determined (N > 7 per condition). Values are shown as mean ± SEM. Asterisk indicates statistical significance (*p* < 0.05) compared to WT. (**f**) Cell lysates of wild type (WT) TRPV5 and the indicated single (W583A, W583Y, W583F) and double (W583A-G579A, W583Y-G579A, W583F-G579A) mutants were immunoblotted with HA antibody, and using β-actin as loading control. A representative immunoblot is shown in the upper panel. The lower panel shows a ^45^Ca^2+^ uptake assay of HEK293 cells transfected with either wild type (WT) TRPV5 or the indicated mutants (N = 9, three independent experiments performed in triplicate). Ruthenium red (RR) is used as control for TRPV5-mediated uptake. Values are shown as mean +/− SEM. Asterisk indicates statistical significance (p < 0.05) compared to WT.

Based on the altered function of the W583 mutants, we hypothesize that the tryptophan serves as channel gate similar to what is observed in some voltage-gated K^+^ channels^28^. Interestingly, prokaryotic K^+^ channel gating is often mediated by glycine helix kinking in combination with an aromatic gate^29^. In TRPV5, a glycine (G579), one α-helical turn above W583, could serve as a flexible hinge point for movement of the lower TM6 helix allowing the W583 residues to adopt different conformations (Fig. 4a). In order to test this hypothesis, G579 was mutated into the more rigid alanine residue. The Ca^2+^ permeability of G579A is significantly reduced (100 ± 2% and 41 ± 3% for wild type and G579A mutant, respectively) as demonstrated by a ^45^Ca^2+^ uptake assay (Fig. 4b). Total protein expression was not altered compared to wild type (Fig. 4b). In addition, cell surface biotinylation assays showed that TRPV5 G579A is present at the plasma membrane, albeit at slightly reduced levels (100 ± 4% and 72 ± 16% for wild type and G57A mutant, respectively) (Fig. S3A). Whole-cell patch clamp recordings demonstrated a significantly diminished current density for G579A, with no difference in the shape of the current/voltage relationship or the EGTA-sensitive current (Fig. 4c,d and Fig. S3B). Cell-attached single channel analysis of the G579A mutant revealed a decreased open probability (3.0 ± 0.6% and 21 ± 4% for G579A and wild type, respectively) (Fig. 4e). Of note, the amplitude of opening was not significantly altered (Fig. 4e, 5.5 ± 0.3 pA for wild type vs 4.8 ± 0.9 pA for G579A). The G579A mutation renders the channel less functional and we hypothesized that it could reduce the severe phenotype of the W583 mutants.

To investigate whether G579A is able to rescue the cell death caused by mutation of W583, three TRPV5 double mutants (G579A-W583Y, G579A-W583F, G579A-W583A) were generated. Interestingly, TRPV5 protein expression of the double mutants containing the aromatic substitutions (G579A-W583Y and G579A-W583F) was noticeably enhanced compared to the single W583 mutants, whereas the phenotype of W583A could not be rescued by addition of the G579A mutation (Fig. 4f, upper panel). Though it seems that Ca^2+^ permeability was not restored, as the ^45^Ca^2+^ uptake was still significantly reduced compared to wild type (WT) TRPV5, similarly as the single W583 mutants (Fig. 4f, lower panel).

### Linking the carboxy-terminus and the pore

**Figure 5:**
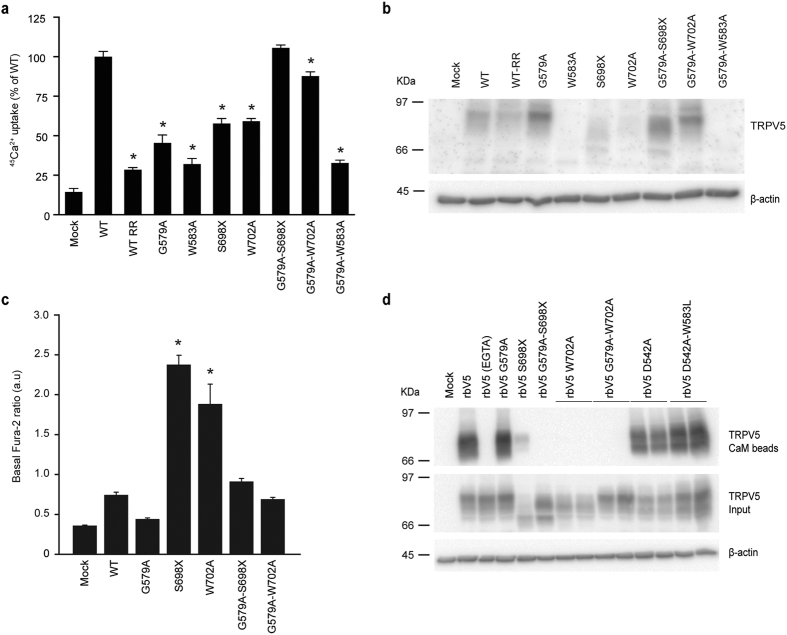
Rescue the TRPV5 CaM binding mutants by the G579A mutation. (**a**) ^45^Ca^2+^ uptake assay of HEK293 cells transfected with either wild type (WT) TRPV5 or the indicated mutants (G579A, W583A, S698X, W702A, G579-W583A, G579A-S698X, and G579A-W702A), depicted as percentage of WT (upper panel) (N = 9, three independent experiments performed in triplicate). Ruthenium red (RR) is used as a negative control. (**b**) Cell lysates of the respective experiments were immunoblotted with HA antibody, and using β-actin as loading control. A representative immunoblot is shown. (**c**) The basal intracellular Ca^2+^ levels are shown as fura-2 ratio in arbitrary units (a.u.) for HEK293 cells expressing either wild type (WT) TRPV5 or the indicated mutants (N = 11–66, total from two independent experiments). Values are shown as mean ± SEM. Asterisk indicates statistical significance (*p* < 0.05) compared to WT. (**d**) CaM binding assay of HEK293 cells transfected with wild type (WT) or indicated mutants of TRPV5. Samples were analyzed by immunoblotting with HA antibody. The CaM beads fraction represents the TRPV5 bound to the CaM agarose beads (top panel) and input demonstrates TRPV5 expression in total cell lysates (bottom panel). Representative immunoblot of three independent experiments is depicted.

Removal of the TRPV5 C-terminal domain (S698X) or ablating its CaM binding site (W702A) is also known to result in enhanced Ca^2+^ influx as a result of diminished CaM-mediated Ca^2+^-dependent channel inactivation^15^,^16^. Hence, we aimed to investigate whether there is a structural crosstalk between the C-terminus and the pore. The G579A mutation was introduced in the TRPV5 S698X and W702A mutants. Protein expression of both the G579A-S698X and G579A-W702A mutants was increased compared to their single mutant counterparts (Fig. 5b). Moreover, it normalized TRPV5 function since ^45^Ca^2+^ uptake levels of both double mutants were comparable to wild type, while the single mutants demonstrated significantly lower ^45^Ca^2+^ uptake due to the reduced cell survival (Fig. 5a). Protein expression and function could not be restored in the G579A-W583A mutant. In line with previous findings, the basal intracellular Ca^2+^ levels were significantly increased in the TRPV5 S698X and W702A mutants^15^,^17^ and we now demonstrated that addition of G579A to these mutants also led to normal Ca^2+^ levels as measured using fura-2-AM (Fig. 5c). Interestingly, this seems independent from CaM binding as functionalized CaM agarose beads were able to bind wild type TRPV5 and the G579A mutant, but not S698X and W702A single or G579A-S698X and G579A-W702A double mutants (Fig. 5d). Of note, binding to CaM beads occurred only in the presence of Ca^2+^, as EGTA-containing buffer will prevent CaM interaction (Fig. 5d). The D542A and D542A-W583L single and double mutants were used as controls to demonstrate that mutation of W583 does not affect the CaM interaction.

## Discussion

This study presents a novel residue (W583) in TRPV5, which affects channel gating and is postulated as an aromatic gate that functions as the final determinant in Ca^2+^ permeation through the channel. Structural movement of this lower gate could be mediated by a nearby glycine hinge (G579), which in turn might play a role in CaM-dependent channel inactivation. Based on our TRPV5 homology model, the rotameric conformations of W583 were analyzed and three major side chain positions were detected: pointing down, pointing up or facing each other. Often, aromatic residues are localized at the interfacial regions of a TM α-helix and the solvent. These residues have a role in anchoring proteins into the cell membrane through interactions with the lipid head groups^30^. In contrast, the current TRPV5 model shows that W583 is located at the intracellular face and it is pointing away from the lipids. This observation, together with the present findings, suggests that W583 does not participate in the interaction of the α-helix with lipids, but may play a role in channel gating. As a result of their size and shape, a small rotation in the aromatic residue can lead to a large change in width of the gate^28^. When the W583 side chains are facing each other, the selective passage of Ca^2+^ is hampered since the estimated pore diameter in this conformation is ~0.8 Å and dehydrated Ca^2+^ has a diameter of 0.99 Å^31^. Upon conformational changes, the side chains can be placed into the other rotameric positions, widening the gate radius to ~11.2 Å, and thereby allowing Ca^2+^ passage. When we characterize the ion conduction pathway of our TRPV5 model, we find that the conserved I575 does not form a constriction point contributing to the lower gate. In contrast, the homologous I679 in TRPV1 forms a constriction (5.3 Å) that blocks ion permeation in the closed state^18^,^32^. Also M578, which is the homologue of the postulated lower gate (M577) in TRPV6, was not identified as major contribution to pore constriction in our model. To approve our data in the context of the recently resolved TRPV6 structure, we have also generated a TRPV5 homology model based on TRPV6 structure (Fig. S2D,E). In both models, we see that W583 lies at the intracellular mouth of the ion conduction pore, and propose that by rotation of its side chain into the ion conduction pathway could form a tight constriction. Homology modeling is a valuable method for structure prediction in the absence of a resolved protein structure, but it is important to remain cautious for potential inaccuracies. Our conclusions about the roles of amino acids are qualitative and not quantitative. Energy refinement methods during model building might force the channel into artificial positions. Hence, the homology model serves as supportive structural material for our experimental data that highlight the importance of W583 in TRPV5 channel function.

A gating function for aromatic residues has been studied in other ion channels. The proton-selective influenza virus M_2_ channel is regulated by a so-called tryptophan gate^33^. This tryptophan gate is stabilized by aspartate residues through a network of hydrogen bonds, which support the closed state conformation of the channel^29^. Upon lowering the outside pH, four histidine residues in this network are protonated, leading to a conformation change of the gate. It has been described for the prokaryotic K^+^ channel KcsA that step-wise conformational changes occur at F103, as a function of channel opening. The position of the side chain of F103 changes upon inner helix movement and is associated with opening of the upper activation gate^34^. Moreover, mutations at this phenylalanine gate have a size-dependent effect on the gating kinetics. Small side-chain substitutions result in impaired inactivation kinetics, while substitution with larger side chain residues has minor effects on channel characteristics^34^. In line with this finding, our study demonstrated that substitution of W583 with a non-aromatic residue (W583Q, W583L, and W583A) results in massive ^45^Ca^2+^ influx and cell death, while change to an aromatic residue (W583F and W583Y) have a milder phenotype. Interestingly, the channel’s open probability of W583F is decreased compared to wild type TRPV5. It should be noted that cell-attached currents are recorded in Ca^2+^-free solution containing Na^+^ as charge carrier. Our hypothesis is that the side chain of the phenylalanine can still close the channel gate to some extent as apparent from the dwell time analysis, thereby allowing passage of the smaller Ca^2+^ ions but rendering the channel less permeable for Na^+^.

Several mechanisms have been proposed for the gating of voltage-gated K^+^ channels^28^: (i) rotation of the inner pore helix around the pore axis, (ii) kinking of the inner pore helix, and (iii) helix kinking in combination with an aromatic gate. Most eukaryotic K^+^ channels are regulated by kinking of the inner pore helix at a proline-valine-proline (PVP) sequence^35^, a motif that is absent in TRPV5. Prokaryotic K^+^ channel gating is often mediated through an aromatic gate in combination with helix kinking by a flexible hinge point at the bottom of the α-helix that is composed of a glycine^28^,^35^. The prokaryotic K^+^ inwardly rectifying KirBac 1.1 channel functions through four phenylalanine residues at the cytoplasmic end of the pore^36^, which are displaced by bending a so-called gating hinge (a glycine) in the inner pore helices, thereby allowing channel opening^28^,^37^. A similar gating mechanism has been proposed for the closely related KirBac 3.1 channel, using a tyrosine residue^38^. The current TRPV5 homology model reveals a highly conserved glycine (G579), located one α-helical turn above the aromatic gate that may produce such a helix kink. Mutation of G579 into an alanine completely blocked the ^45^Ca^2+^ uptake by reducing the open probability, leading to the hypothesis that G579 is essential for proper channel gating. Substitution of this residue is suggested to hold the channel into a closed state. On contrary, mutation to proline could potentially bend the helix and keep the channel open, however, a rather flickering single channel behavior was observed (data not shown).

Importantly, future studies will aim at determining the 3D structure of TRPV5 to resolve this potentially unique pore and provide further insight into channel gating mechanisms. A key question is which stimuli trigger the conformational change that precedes channel gating. For example, the TRPV5 TM6 α-helix changes its conformation upon extracellular acidification^39^. In addition, the pore helix is rotated along its axis as a result of intra- or extracellular acidification^40^. A pH-sensitive histidine (582) is located directly above the tryptophan gate. Protonation of the histidine residue might cause the hinge to change the relative positions of the tryptophan residues, thereby opening or closing the channel. In addition, studies on other ion channels have suggested a link between the channel pore and C-terminal regulatory regions via transducing conformational changes to the channel gate^41^,^42^,^43^. External factors such as phosphatidylinositol 4,5-bisphosphate (PIP_2_) can potentially regulate the TRPV5 gating mechanism via binding to the TRP domain in the C-terminus^44^. PIP_2_ stabilizes TRPV5 in its open conformation^45^. Interestingly, TRPV5, TRPV6 and the K^+^ channels, which display a similar gating mechanism, are all stimulated by PIP_2_^28^,^46^. In contrast, TRPV1 shows no conservation of the tryptophan gate and is inhibited by PIP_2_^44^. Our study now demonstrates a link between CaM binding and the channel pore, since the G579A mutation rescues the Ca^2+^ overloading phenotype of the CaM-binding mutants W702A and G698X. Alternatively, loss of CaM binding may transform the inactive G579A mutant into a functional channel through structural rearrangements. Hence, we suggest that C-terminal CaM binding is an essential component of TRPV5 gating by inducing a conformational change of the channel pore and initiating Ca^2+^-dependent channel inactivation. We were unable to test this hypothesis with our current model as the corresponding region was unresolved in the TRPV1 structure. Future TRPV5 models and structures may facilitate viewing this region of the protein and assessing interactions between TM6 and the CaM binding domain.

In conclusion, our study demonstrates a unique mechanism of channel gating for TRPV5 and provides novel insight into TRP channel structure-function relationship, as the tryptophan gate is not conserved in other TRPV family members.

## Methods

### Buffers

Lysis buffer for cell lysis contained 50 mM Tris-HCl (pH 7.5), 150 mM NaCl, 1 mM EDTA, 1 mM EGTA, 1% (v/v) Triton X-100, 1 mM sodium orthovanadate, 10 mM Na-β-glycerophosphate, 50 mM Na^+^ fluoride, 10 mM Na^+^ pyrophosphate, 0.27 M sucrose, and the protease inhibitors 1 mM phenylmethanesulfonylfluoride (PMSF), 10 μg/ml leupeptin, 10 μg/ml pepstatin A, 5 μg/ml aprotinin. Buffer A contained 50 mM Tris-HCl (pH 7.5) and 0.1 mM EGTA. TBS-Tween (TBS-T) was Tris-HCl (pH 7.5), 0.15 M NaCl and 0.1% (v/v) Tween-20. Laemmli sample buffer (5X) contained 250 mM Tris-HCl pH 6.8, 10% (w/v) SDS, 30% (v/v) Glycerol, 5% (v/v) β-mercaptoethanol, and 0.02% (w/v) bromophenol blue. KHB buffer for ^45^Ca^2+^ uptake contained 110 mM NaCl, 5 mM KCl, 1.2 mM MgCl_2_, 0.1 mM CaCl_2_, 10 mM Na-acetate, 2 mM NaH_2_PO_4_, and 20 mM HEPES (pH 7.4, NaOH). Stop buffer for ^45^Ca^2+^ uptake contained 110 mM NaCl, 5 mM KCl, 1.2 mM MgCl_2_, 10 mM Na-acetate, 0.5 mM CaCl_2_, 1.5 mM LaCl_3_, and 20 mM HEPES (pH 7.4, NaOH).

### Generation of a TRPV5 homology model

The YASARA^47^ & WHAT IF^48^ Twinset was used to build a homology model of TRPV5, based on the TRPV1 and TRPV6 structures as a modeling template (29% sequence identity, PDB files 3j5p and 3j5q^18^,^32^, 60% sequence identity, PDB file 5iwk^19^). An automatic script with standard parameters was used. The model contained residue 1–644 of rabbit TRPV5. YASARA was used for detailed evaluation of the models.

The generated homology model was further visualized in COOT^49^ and pore radii were analyzed using the HOLE program^50^.

### Cell culture

HEK293 cells were purchased from ATCC (LGC Standards GmbH, Wesel, Germany) and grown in Dulbecco’s modified eagle’s medium (DMEM, Bio Whittakker-Europe, Verviers, Belgium) containing 10% (v/v) fetal calf serum (PAA, Liz Australia), 2 mM L-glutamine and 10 μg/ml non-essential amino acids at 37 °C in a humidity-controlled incubator with 5% (v/v) CO_2_. HEK293 cells were transiently transfected with the respective DNA construct using polyethyleneimine (PEI, Brunswig/Polysciences Inc) with a DNA:PEI ratio of 1:6 for the Fura-2 imaging and all biochemical experiments. For the patch clamp experiments, plasmids were transiently transfected using lipofectamine 2000 (Invitrogen, Breda, The Netherlands) in a DNA:lipofectamine ratio of 1:2. Cells were used 24–36 hours after transfection.

### DNA constructs

The pCINeo/IRES-GFP plasmid encompassing HA-tagged rabbit TRPV5 was generated as described previously^14^,^51^. Restriction enzyme digestions, DNA ligations and other recombinant DNA procedures were performed according to standard protocols. Mutations were introduced using the QuikChange site-directed mutagenesis method (Stratagene, La Jolla, USA), following the manufacturer’s protocol. All constructs were verified by DNA sequencing.

### Cell lysis and immunoblotting

Cells were disrupted in ice-cold lysis buffer, and the lysates were clarified by centrifugation at 4°C for 15 minutes at 16,000 g. Protein concentration was measured using the Bradford method, according to standard protocol. Cell lysates in Laemmli sample buffer were subjected to 8% (w/v) SDS-PAGE, transferred to polyvinylidene fluoride (PDVF) membranes. Membranes were blocked for 30 minutes with 5% (w/v) non-fat dry milk (in TBS-T) and immunoblotted overnight at 4 °C using anti-HA antibody (1:5,000, Cell Signalling Technology, Beverly, MA, USA) or anti-beta-Actin (1:10,000, Sigma A5441). Subsequently, the blots were washed with TBS-T, incubated with secondary peroxidase-labelled goat anti-mouse IgG (1:10,000, Chemie Brunschwig, Basel, Switzerland) for 1 hour at room temperature. After subsequent washes, they were visualized with enhanced chemiluminescence reagent using the Bio-Rad ChemiDoc XRS imaging system.

### Cell surface biotinylation assay

One day after transfection, HEK293 cells were reseeded on poly-L-lysine (Sigma, St Louis, MO, USA) coated (0.1 mg/ml) 6-well plates. Subsequently, cells were biotinylated as described previously^52^ and disrupted in 0.5 ml lysis buffer. Lysates were clarified by centrifugation at 4 °C for 15 minutes at 16,000 g and the biotinylated proteins were precipitated via incubation with neutravidin beads (Pierce, Ettenleur, the Netherlands) for 2 hours at 4 **°**C. The precipitates were washed three times with lysis buffer and two times with buffer A. After elution by 2X Laemmli sample buffer, TRPV5 protein expression at the cell surface and in total cell lysate was determined by immunoblotting using anti-HA antibody.

### CaM binding assay

The lysis buffer for cell lysis contained either 1 mM Ca^2+^ or 5 mM EGTA (negative control). Following, 1 mg of clarified cell lysate was incubated with CaM agarose beads (Sigma) for 2 hours at 4 °C under gentle agitation. Subsequently, the immunoprecipitates were washed three times with lysis buffer containing 1 mM Ca^2+^ or 5 mM EGTA and then twice with Buffer A. Proteins were eluted by re-suspending the immunoprecipitates in 30 μl of 2X Laemmli sample buffer.

### ^45^Ca^2+^ uptake assay

One day after transfection, HEK293 cells were reseeded on poly-L-lysine coated (0.1 mg/ml) 24-well plates. Radioactive ^45^Ca^2+^ uptake was performed as previously described^52^. In short, cells were pretreated for 30 min with 25 μM BAPTA-AM at 37 **°**C, subsequently washed twice with warm KHB buffer, and then incubated for 10 min at 37 **°**C with ^45^Ca (1 μCi/ml) in KHB buffer containing the voltage-gated Ca^2+^ channel blockers felodipine (10 μM) and verapamil (10 μM). Ruthenium red (10 μM) was used for blocking the TRPV5-mediated ^45^Ca^2+^ uptake. After 10 min, the cells were washed three times with ice-cold stop buffer, and lysed in 0,05% SDS. This was transferred to tubes containing scintillation solution and the amount of ^45^Ca was measured using a scintillation counter.

### Intracellular Ca^2+^ measurements using fura-2-AM

Cells were seeded on fibronectin (Roche, Indianapolis, USA) coated coverslips (diameter 25 mm) and transfected with the appropriate TRPV5 constructs. The next day, cells were loaded for 20 minutes with 3 μM fura-2-acetoxymethyl ester (fura-2-AM; Molecular Probes) and 0.01% (v/v) Pluronic F-129 (Molecular Probes) in Krebs solution (5.5 mM KCl, 147 mM NaCl, 1.2 mM MgCl_2_, 1.5 mM CaCl_2_, 10 mM glucose, and 10 mM HEPES/NaOH, pH 7.4) at 37 °C. Subsequently, the cells were washed once with Krebs solution and allowed to equilibrate at 37 °C for another 10 minutes. Details of microscopy procedures and quantitative image analysis have been described previously^17^. In short, fura-2-loaded cells were placed on an inverted microscope using an incubation chamber containing Krebs solution and intracellular Ca^2+^ levels were calculated from the fluorescence emission ratio of 340 and 380 nm excitation. Our data shows basal Ca^2+^ levels that were measured immediately at t = 0, the start of the experiment. All measurements were performed at room temperature.

### Electrophysiology

All patch clamp recordings were acquired using an EPC-9 amplifier and the Patchmaster software (HEKA electronics, Lambrecht, Germany). The sampling interval was set to 200 μs for whole-cell recordings and 100 μs for single-channel recordings. A low-pass filter set at 3.6 kHz was used for whole-cell recordings, and single-channel recordings had a low-pass filter set at 5 kHz. The Humbug 50/60 Hz noise eliminator (Quest Scientific, Vancouver, Canada) was used for reduction of electrical noise. Pipettes for whole-cell recordings were pulled from thin-walled borosilicate glass (Harvard Apparatus, March-Hugstetten, Germany) and had resistances between 1 and 3 MΩ. Series resistance compensation was set to 75–95% in all whole-cell experiments. Pipettes for single-channel recordings were pulled from thick-walled borosilicate glass (Harvard Apparatus) and had resistances between 8 and 11 MΩ. The extracellular solutions for whole-cell recordings were: 1) nominally divalent-free (nDVF) solution containing (in mM) 150 NaCl, 6 CsCl, 10 glucose, 10 HEPES/NaOH, pH 7.4; and 2) divalent-free (DVF) solution, which is nDVF solution with addition of 100 μM EGTA. Hence, the EGTA-sensitive whole-cell current was obtained by perfusion of DVF. The pipette solution is composed of (in mM) 20 CsCl, 100 CsAspartate, 1 MgCl_2_, 10 BAPTA, 4 Na_2_ATP, 10 HEPES/CsOH, pH 7.2. A voltage step protocol, consisting of voltage steps from −100 to +40 mV, was applied from a holding potential of +20 mV. The extracellular solution for cell-attached measurements contained (in mM) 150 NaCl, 6 CsCl, 10 glucose, 10 HEPES/NaOH, pH 7.4. The pipette solution comprised (in mM) 140 NaCl, 10 EGTA, 10 HEPES/NaOH, pH 7.2. Single channel activity was monitored upon a 10 s step to −80 mV from a holding potential 0 mV. All experiments were performed at room temperature. The analysis and display of whole-cell recordings were performed using Igor Pro software (WaveMetrics, Lake Oswego, USA). Single channel recordings and dwell time analysis were analyzed using the Qub software package (www.qub.buffalo.edu). The distribution of the channel open and closed states was fitted to an exponential fit and he time constant of each exponential is related to the rate of kinetic state transitions.

### Trypan blue assay

Two days after transfection, cells were resuspended in phosphate buffered saline (PBS), pH 7.4, adding 4% (w/v) trypan blue (Sigma) to a final concentration of 0.08% (w/v). The number of non-viable cells was counted using a hemocytometer. Cell death is measured as the number of non-viable cells divided by the total number of cells within the grids on the hemocytometer.

### Ethics statement

Research was carried out in accordance with the relevant guidelines and regulations of the Radboud Institute for Molecular Life Sciences, Radboudumc. Experimental protocols were approved by the Radboud Institute for Molecular Life Sciences, Radboudumc.

### Statistical analysis

All data are shown as mean ± standard error of the mean (SEM). Statistical significance (*p* < 0.05) was determined by a one-way ANOVA and a Dunnett Multiple Comparison post-hoc test. Data were analyzed using Prims 6 software (Graphpad Software Inc, La Jolla, CA, USA).

## Additional Information

**How to cite this article:** van der Wijst, J. *et al*. A Gate Hinge Controls the Epithelial Calcium Channel TRPV5. *Sci. Rep.* **7**, 45489; doi: 10.1038/srep45489 (2017).

**Publisher's note:** Springer Nature remains neutral with regard to jurisdictional claims in published maps and institutional affiliations.

## Supplementary Material

Supplementary Information
