# Supplementary material for: A Gate Hinge Controls the Epithelial Calcium Channel TRPV5
Source: Sci Rep. 2017 Apr 4;7:45489. doi: 10.1038/srep45489 (PMC5379628; doi:10.1038/srep45489)
Supplement: Supplementary Information [file srep45489-s1.pdf]

A Gate Hinge Controls the Epithelial Calcium Channel TRPV5

Jenny van der Wijst[1], Elizabeth H.P. Leunissen[1], Maxime G. Blanchard[1], Hanka Venselaar[2], Sjoerd Verkaart[1], Candice E. Paulsen[3], René J.M. Bindels[1], Joost G.J. Hoenderop[1]

[1]Department of Physiology, [2]Centre for Molecular and Biomolecular Informatics, Radboud Institute for Molecular Life Sciences, Radboud university medical center, Nijmegen, The Netherlands,

[3]Department of Physiology, University of California, San Francisco, California 94158-2517, USA.

Supplemental Information

Supplemental Figure legends

Figure S1, related to Figure 2 - Single channel activity of the TRPV5 W583 mutants. (A) Representative traces of cell-attached single channel recordings of W583A (top), W583Y (middle), W583Q (bottom). The average open probability (B) and amplitude (C) are plotted for wild type (WT) and the indicated mutants (N>7 per condition). Values are shown as mean ± SEM. Asterisk indicates statistical significance ($p<0.05$) compared to WT. (D) The rate constants (k) of switching between the open (O) and two closed states (C1 and C2) are extracted by two-exponential fit of the model-based distribution of dwell times. Average k values of wild type (WT) TRPV5, W583F, and W583L are shown (N>7).

Figure S2, related to Figure 3 - Homology model of TRPV5. (A) The tetrameric TRPV5 structure is depicted in top (left panel) and bottom (right panel) view. Each monomer is color-coded. (B) A detailed top view on the pore of the channel shows the selectivity filter at the outer surface, which is composed of four aspartic acid residues (D542) in green (left panel). At the inner surface of the pore, the tryptophan residues (W583) are depicted in yellow. (C) A front close-up view of the tetrameric channel demonstrates that the side chains of W583 (yellow) are sticking towards the permeation pathway. (D) The tetrameric TRPV5 model, based on the TRPV6 structure (5iwk), is depicted in front view. Each monomer is color-coded. (E) The ion permeation pathway of TRPV6 (5iwk) and the TRPV6-based TRPV5 model is mapped with the HOLE program. Residues located at the selectivity filter and lower gate are indicated to depict the upper and lower restrictions.

Figure S3, related to Figure 4 - Expression and function of TRPV5 G579A. (A) Cell surface biotinylation of HEK293 cells transfected with wild type (WT) TRPV5 or G579A. The biotin fraction represents the TRPV5 present at the plasma membrane

(top panel) and input demonstrates TRPV5 expression in total cell lysates (bottom panel). Representative immunoblot of three independent experiments is depicted and quantification of the biotin fraction is shown in the right panel. (B) The I-V relation of mock, wild type (WT) TRPV5 and G579A is extracted through a voltage step protocol in DVF solution.

Figure S1

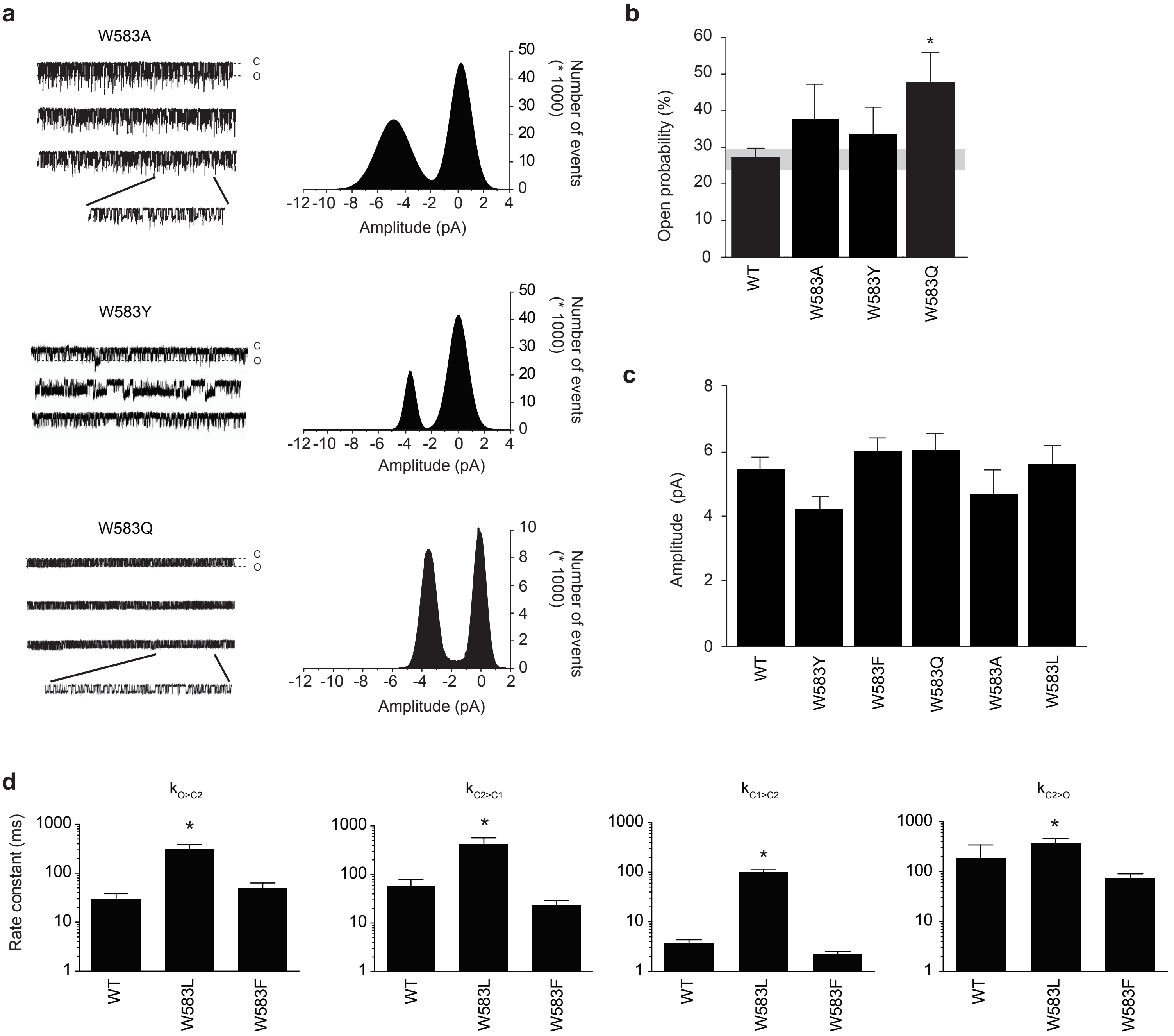
a
W583A
C
O
Number of events (* 1000)
Amplitude (pA)
W583Y
C
O
W583Q
C
O
b
Open probability (%)
WT
W583A
W583Y
W583Q
*
c
Amplitude (pA)
WT
W583Y
W583F
W583Q
W583A
W583L
d
$k_{O>C2}$
$k_{C2>C1}$
$k_{C1>C2}$
$k_{C2>O}$
Rate constant (ms)
WT
W583L
W583F
*

Figure S2

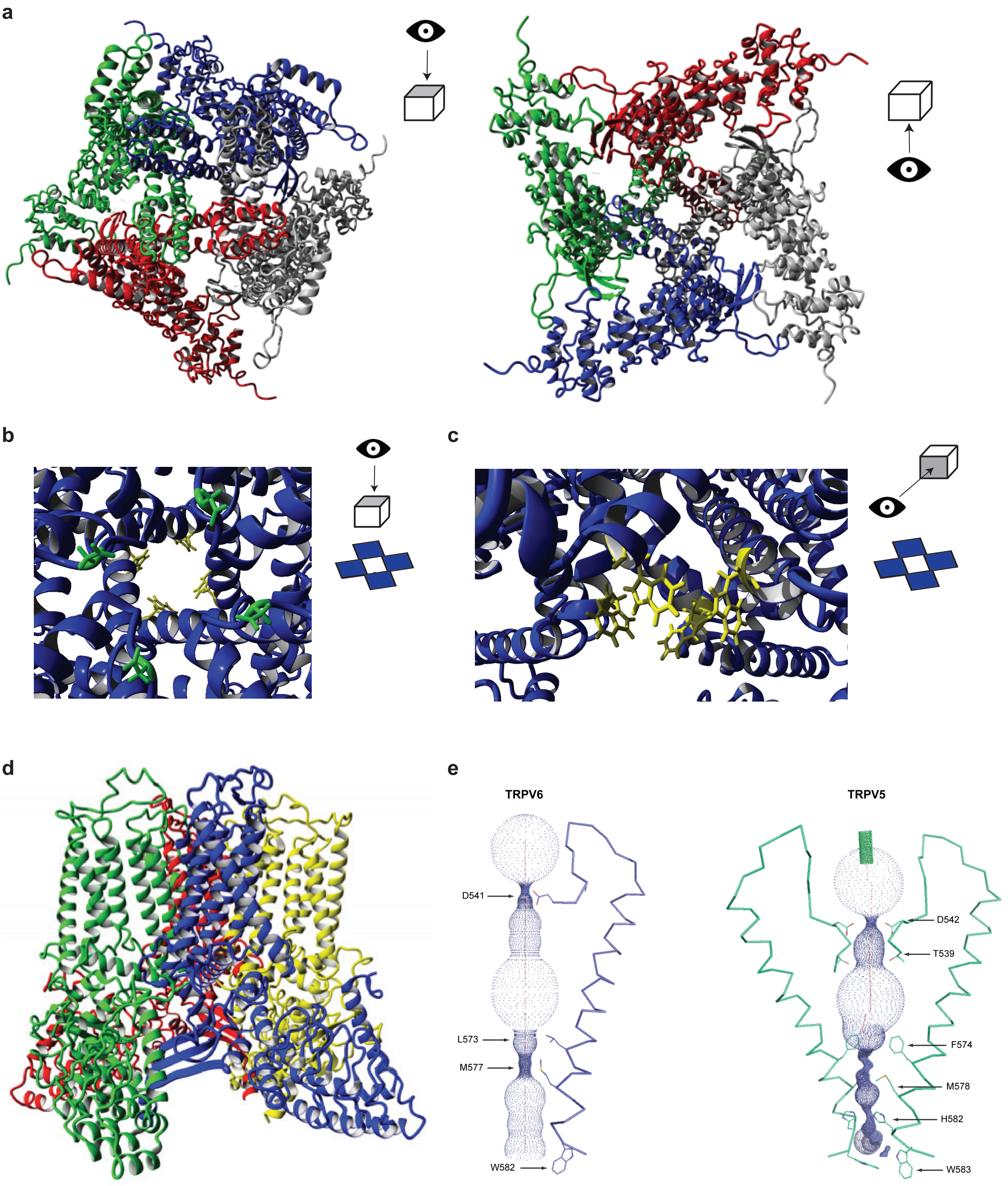

a
b
c
d
e
TRPV6
TRPV5
D541
L573
M577
W582
D542
T539
F574
M578
H582
W583

Figure S3

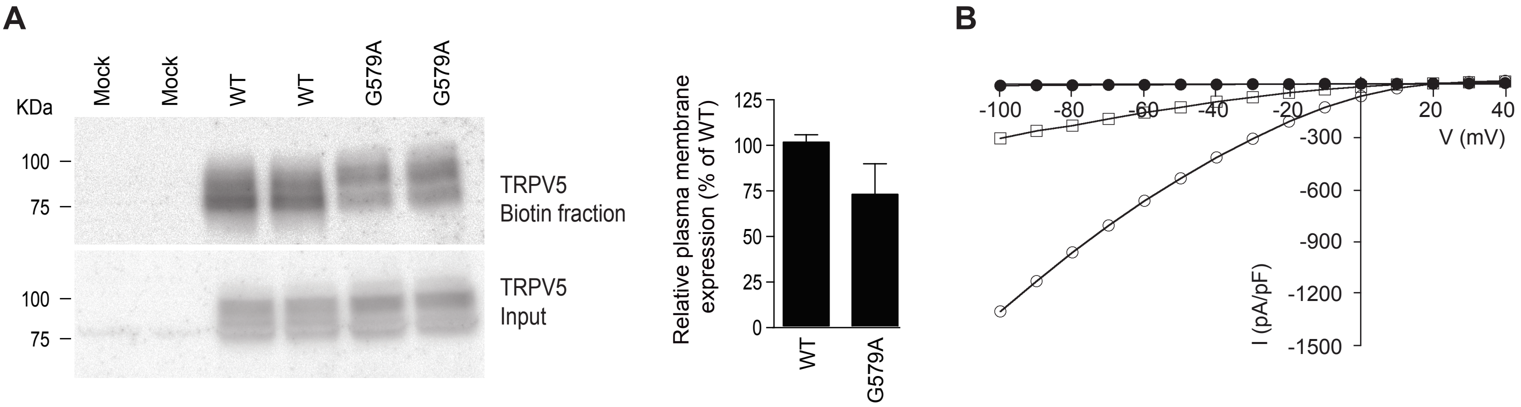
A
Mock
Mock
WT
WT
G579A
G579A
KDa
100
75
TRPV5
Biotin fraction
100
75
TRPV5
Input
Relative plasma membrane expression (% of WT)
125
100
75
50
25
0
WT
G579A
B
-100
-80
-60
-40
-20
20
40
V (mV)
-300
-600
-900
-1200
-1500
I (pA/pF)
